# A Morphometric Evaluation of the Mandibular Condyle, Coronoid Process, and Gonial Angle: Age and Gender Differences in CBCT Imaging

**DOI:** 10.3390/diagnostics15121459

**Published:** 2025-06-08

**Authors:** Mehmet Emin Dogan, Burcu Nur Turkoglu, Ilhan Şengul

**Affiliations:** 1 Faculty of Dentistry, Department of Dentomaxillofacial Radiology, Harran University, Sanliurfa 63200, Türkiye; turkogluburcunur@gmail.com; 2 Şanlıurfa Oral and Dental Health Hospital, Sanlıurfa 63010, Türkiye; dtilhansengul@yandex.com

**Keywords:** cone beam CT, mandible, image analysis, clinical diagnosis

## Abstract

**Background:** It has been suggested that the mandible may differ according to gender. The fact that the mandible and the anatomical structures on it show various changes with age and gender differences is important in gender and age determination. The aim of this study was to evaluate the morphometric variation in the condyle and coronoid processes and the gonial angle, which are anatomical structures forming the mandible, with gender and age. **Methods:** Cone beam CT images of 141 individuals (78 female, 63 male) were used in this study. The images of all patients were obtained with the same X-ray device; the images were obtained at 70 kVp, 10 mA, and a 32 s exposure time in accordance with the manufacturer’s recommendations. Images displayed with 0.3 mm voxel with the IRYS 15.0 program were examined in the axial, sagittal, and coronal planes, and measurements were taken. The gonial angle, coronoid notch, condyle height, condyle–coronoid distance, and sigmoid notch depths were evaluated. **Results:** The average age of 141 individuals was 43.21 ± 15.96 years, and 55.3% of them were female and 44.7% were male. The mean right gonial angle in females (128.66 ± 5.50°) was significantly higher than in males (125.68 ± 5.10°) (*p* < 0.005). Similarly, the mean left gonial angle in females (128.84 ± 5.97°) was significantly higher than in males (125.26 ± 4.89°) (*p* < 0.005). The sigmoid notch depth was found to be greater in men, with an average of 13.88 ± 2.46 mm, while in women, it had an average of 13.13 ± 1.80 mm, and this difference was statistically significant (*p* < 0.005). The relationship between the two sides’ coronoid notch height, sigmoid notch depth, condyle height, and condyle–coronoid distance and age groups was not statistically significant (*p* > 0.005). **Conclusions:** Overall, our findings indicate that the male mandible may have a longer condyle, a narrower gonial angle, and a wider sigmoid notch depth than that of females. It has been observed that ramus measurements such as condyle length and sigmoid notch depth may be important in gender discrimination, and the male mandible exhibits larger values in these parameters. No differences in parameters were observed between age groups.

## 1. Introduction

In the craniomaxillofacial complex, muscles and bones are anatomically and functionally interconnected to perform the function of movement [1,2]. The human mandible in this complex plays a vital role in various functions such as chewing, speech, and supporting the facial structure. Anatomically, it contains key structures such as the condyle, coronoid process, and sigmoid notch. These structures not only aid in the movement function of the jaw but also contribute to the overall biomechanical balance of the temporomandibular joint (TMJ) and the craniomaxillofacial complex [2]. According to Wolff’s law, a bone’s structure and morphology show adaptive changes depending on the mechanical loads and muscle forces applied to it. When the bone is exposed to mechanical stress, the osteoclastic activity increases in the load area, while osteoblastic activity increases on the opposite side [3]. The mandible is one of the bones that is exposed to significant biomechanical stresses by the surrounding muscle tissues. The application of these stresses to the bone over the years can cause many changes in the mandible with age [4,5,6]. On the other hand, studies have reported that muscle strength is affected by gender differences, suggesting that changes in the mandible may differ according to gender [7,8]. Therefore, it is normal for the mandible and the anatomical structures on it to show various changes with age and gender differences [4,5,6]. The gonial angle is one of the parameters that are used in growth direction analyses in orthodontics and in determining gender and age in forensic medicine [8]. As it continues superiorly from the ramus of the anatomical mandible, two protrusions are seen. These protrusions, called the condyle and coronoid, are involved in many functions, such as opening and closing the mouth [9].

In the literature, various techniques have been used to examine these structures, such as cone beam computed tomography, multidetector computed tomography (MDCT), panoramic radiography, and cephalogram, both using cone beam computed tomography and direct measurement on dry bones. In this study, cone beam CT was preferred due to its low radiation dose, 3D imaging, and success in measuring length [8]. The aim of the study is to evaluate the morphometric variation that occurs in the condyle, coronoid processes, and gonial angle that form the mandible with gender and age.

## 2. Materials and Methods

This study was approved by the Harran University Clinical Research Ethics Committee, with the decision number HRÜ/24.20.03. G*Power 3.1.9.7 was used to determine the sample size of the study. When α = 0.05, effect size = 0.5, and 1-β = 0.85 were taken for the independent sample t-test, the sample size was found to be 59 for each group and 118 in total. In this study, 451 images were examined from the Harran University Oral, Dental and Jaw Radiology archives; images with maxillofacial deformity, craniofacial operation or trauma findings, or insufficient image quality were not included in the study. Cone beam CT images of 141 individuals (78 female, 63 men), taken with the Castellini Xradius Trio Plus (Imola, Italy) device, were used in the study. Individuals aged between 20 and 74 were divided into 5 groups: 20–29 years, 30–39 years, 40–49 years, 50–59 years, and 60 and over.

The images of all patients were obtained with the same cone beam CT device. Images were obtained at 70 kVp, 10 mA, and 32 s of exposure time in accordance with the manufacturer’s recommendations. In order to avoid positioning errors as much as possible, the image acquisition and calibration were performed by a single technician, and ideal images were obtained by adhering to the reference points determined by the manufacturer on the device. Images viewed with 0.3 mm voxel with the IRYS 15.0 program were examined and evaluated in the axial, sagittal, and coronal planes.

**Figure 1:**
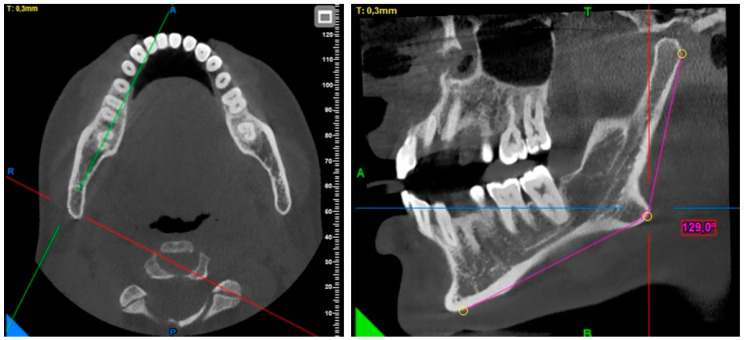
Gonial angle measurement.

**Gonial angle measurement:** The menton point was determined by checking from the sagittal, axial, and coronal planes. In the sagittal plane, the gonial angle was measured at the intersection of the line drawn from the gonion point to the posterior of the condyle and the line drawn to the menton point (Figure 1).

**Figure 2:**
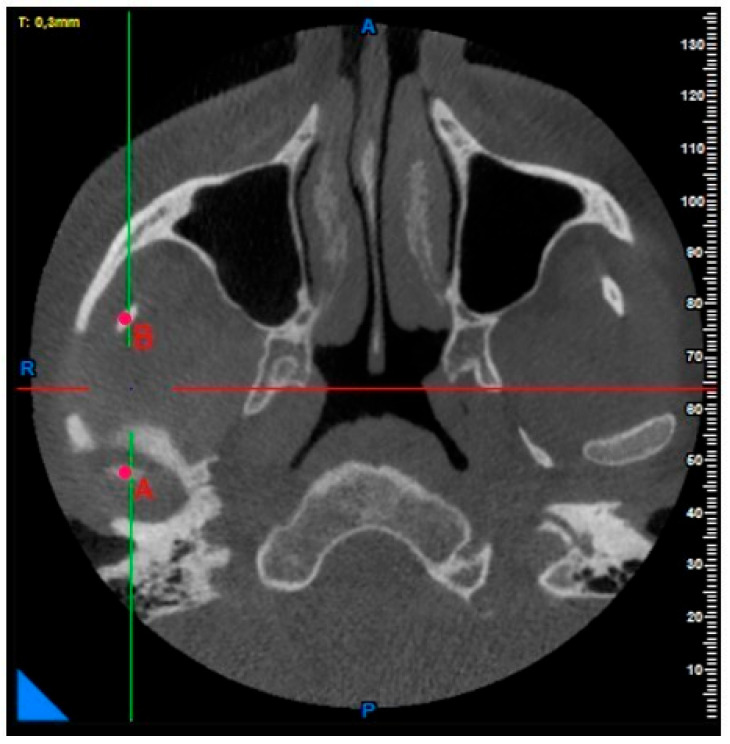
Point A: apex of condyle; Point B: apex of coronoid notch.

**Figure 3:**
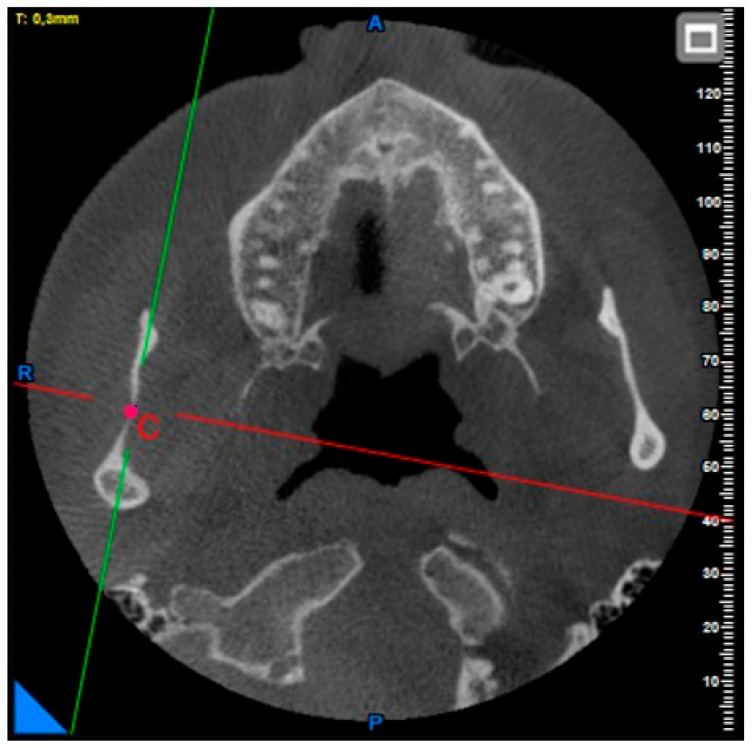
Point C: the deepest point of the sigmoid notch.

**Coronoid notch, condyle height, condyle–coronoid distance, and sigmoid notch depth measurements:** After determining the top points of the condyle and coronoid notch in the axial plane, the deepest point of the sigmoid notch was determined in the same plane (Figure 2 and Figure 3).

**Figure 4:**
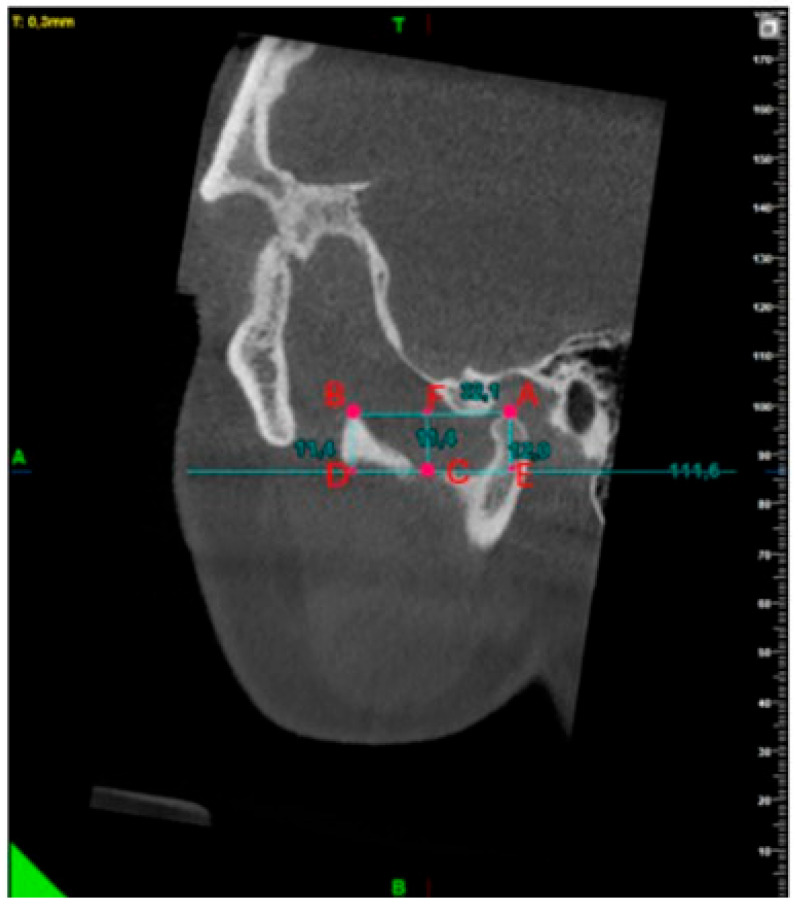
Measurement of condyle, coronoid processes, sigmoid notch depth, and condyle–coronoid distance. |AE| Condyle height;|FC| sigmoid notch depth; |BD| coronoid process height; |AB| condyle–coronoid distance.

A tangent line was drawn to the deep point of the sigmoid notch in the sagittal plane based on the study by Gomes et al. [3]. The condyle and coronoid notch heights were measured by dropping perpendiculars from the top points of the condyle and coronoid process to this line. The distance between the condyle and coronoid top points was determined. The notch depth was measured by lowering a perpendicular from the deepest point of the sigmoid notch to the condyle and coronoid plane on the CBCT sagittal section [10] (Figure 4).

**Statistical analysis:** The data was analyzed using the IBM SPSS version 25 (Armonk, NY, USA) package program. It was analyzed separately for the right and left sides in terms of age and gender, and descriptive statistics were used in the analysis of categorical data. The independent sample t-test was used in the distribution according to gender. The ANOVA test was used to examine the distribution according to age groups. The post hoc test was used for multiple comparisons. The Kappa test was used for the calculation of intra-observer reliability.

## 3. Results

The intra-observer reliability was found to be at a strong level (0.89). The average age of the 141 individuals included in the study was 43.21 ± 15.96, and 55.3% of them were female and 44.7% were male. The distribution of measurements by gender is shown in Table 1.

The right gonial and left gonial angle averages were found to be higher in females than in males (*p* < 0.005). Both the right and left condyle height averages were measured to be higher in males than in females (*p* < 0.005). The average sigmoid notch depth was found to be significantly greater in males (*p* < 0.005). The condyle–coronoid distance was found to be significantly wider on the right side in males (*p* < 0.005). There was no significant difference in the right coronoid height according to gender (*p* > 0.005). These difference markers, observed according to gender, provide useful information in both gender determination and orthodontic and jaw surgery planning.

The comparison of age groups is provided in Table 2. When the relationships between age groups and parameters were examined, no statistically significant relationship was observed (*p* > 0.005).

An increase in the gonial angle, a decrease in the coronoid height, and a decrease in the sigmoid notch depth were observed with aging. But this was not statistically significant. The relationship between the two sides’ coronoid notch height, sigmoid notch depth, condyle height, and condyle–coronoid distance and age groups was not statistically significant (*p* > 0.005).

## 4. Discussion

The morphological features of the mandible are shown in the literature as one of the structures that exhibit the most significant sexual dimorphism after the pelvis [8]. In the postnatal period, especially with puberty, the growth patterns of girls and boys differ; since boys develop larger and stronger bone structures and muscle mass after puberty, a thicker mandible structure can be seen in male at later ages [11].

In this study, the relationship between mandibular morphometric parameters and age and gender was examined, and the findings were compared with other studies in the literature. The mandible develops largely under the influence of the masticatory muscles. It has been suggested that excessive masseter and temporal muscle activity may cause the gonial aperture to become smaller by creating a more prominent corner in the lower jaw, but controversial results have been reported in the literature as to whether the gonial angle values differ according to gender [8]. Dutra et al. found that the gonial angle values that they evaluated in panoramic radiography were not statistically significantly related to gender groups [12]. On the other hand, Chloe, Abu Taleb, Ingaleshwar et al. reported significantly lower gonial angles in males [13,14,15].

In their study, Direk et al. evaluated age, gender, and mandibular morphometry in MDCT images and found the gonial angle to be statistically significantly higher in the female group [16]. Additionally, Gamba et al. reported that the gonial angle was lower in females. Gamba et al. found significantly higher gonial angles in males in the gonial angle values that they measured in cone beam CT images [17]. Bulut et al., in their study in which they evaluated the gonial angles of individuals divided into age groups, found that the gonial angle was significantly higher in females in the 60–80 age group, but no statistically significant difference was found in the other groups [18]. It has been frequently reported in the literature that the gonial angle is generally lower in males and higher in females [13,14,15]. A similar trend was observed in our study, and a significant difference was detected between the genders.

Many researchers have evaluated the condyle height and used various reference points to calculate it. In a study conducted by Jyothsna et al., the distance between the condyle and gonion points was measured and found to be greater in male individuals, and the difference was stated to be significant [19]. On the other hand, Mohsen et al. measured the condyle height on the line from the condyle to the sigmoid plane in their study, where they made a three-dimensional evaluation of the mandibular condyle in cone beam CT images. The mean values of the mandibular condyle height and width were more pronounced in males than in females, but they were not statistically significant [20]. Similar findings were obtained in this study. A greater condyle length was found in males, and the difference was statistically significant.

In our study, it was observed that there was no statistical relationship between the coronoid process length and gender. In the literature, there are different results regarding the height of the coronoid process. While some researchers could not detect a significant difference, others found the height of the coronoid process to be greater in male individuals. For example, in a study on dry bone mandibles, the coronoid height was identified as one of the measurements showing the highest sexual dimorphism and was measured to be significantly greater in males [21]. This result suggests that the size of the coronoid process may develop more in males in relation to sex hormones and muscle activity. In contrast, some studies based on radiological measurements have concluded that the length of the coronoid process does not play a significant role in gender differentiation. Aditi Ramesh et al., who measured the condyle–gonion and coronoid–gonion distances radiographically, reported that although the condyle height was significantly greater in males, the coronoid height did not vary according to gender. In the same study, it was particularly emphasized that the coronoid height did not play a significant role and that this finding was consistent with previous studies [22]. Similarly, in a study by Samatha et al., they reported that the coronoid process length did not contribute significantly to gender determination. It was stated that other measurements, such as the condyle height, could provide the main distinction [23].

The inconsistent results in the literature regarding the height of the condyle and coronoid process may be due to differences in measurement techniques and populations. Anthropometric studies that directly measure on dry bone can show coronoid values more clearly, because they are not affected by soft tissue and radiographic distortions. However, when measurements are made using methods such as panoramic radiography, the projection angles or superposition of neighboring structures can reduce the reliability of the measurements. In addition, it has been shown that the development of the coronoid process is closely related to the strength of the temporalis muscle and individual chewing habits, and that the coronoid height undergoes resorption after surgical removal of the temporalis muscle in rats [24]. The coronoid length results in our study are consistent with the literature findings based on radiological methods; no significant difference was found between the sexes. This shows that the coronoid process may not be a reliable sex parameter on its own. However, some studies examining the relationship between the shape of the coronoid process (such as triangular, round, hooked) and sex have reported that the triangular coronoid shape is more frequently encountered in females [25]. Therefore, morphological variations in the coronoid rather than its quantitative length may provide clues about sex.

In our study, gender differences in the coronoid–condyle distance were examined, and it was observed that this could be observed to different degrees on the right and left sides of the mandible. While there was a significant distance difference between the gender groups on the right side, this difference was less pronounced on the left. Although such asymmetric results are rarely encountered in bilateral measurements in the literature, the general asymmetric tendency of the human body and functional habits may be the reason for this situation. Right–left asymmetry in the mandible is mostly associated with the dominant chewing side or unilateral chewing habits of individuals. Adaptive changes may occur on the bone structures on the side where the chewing function is used more frequently. For example, it has been suggested that in individuals who chew unilaterally for a long time, there may be changes in bone morphology as a result of hypertrophy of the muscles on that side and greater loading of the bones. Lyu YS et al., in their studies on cone beam CT images of patients with unilateral chewing habits, found that the height of the condyle on the one-sided chewing side was significantly higher than on the other side [26]. In this case, the distance between the condyle and coronoid may also differ slightly on the working side. On the other hand, complete symmetry is rarely seen during growth and development [27]. The difference in the coronoid–condyle distance between the sides that was observed in our findings may be due to the asymmetric characteristics of the individuals in our sample or to factors affecting the measurement sensitivity. A possible explanation for this situation may be that our study had more right- or left-handed individuals on a certain side, corresponding to the dominant chewing side. As a result, it would not be correct to generalize the difference found in a single study. When the relationships between mandibular parameters and age groups were examined, no statistical relationship was found in our study. Bahşi et al. could not find a statistically significant difference between the gonial angle and age groups in cone beam CT images [8]. On the other hand, Larrazabal-Moron et al. found a negative significant correlation between age and gonial angle values in their study evaluating panoramic X-rays [28]. Assari et al. reported in their study that the condylar height had no significant relationship with age groups [29].

When planning orthognathic surgery, it will be beneficial to plan the incisions to be made in the chin differently according to gender, considering the parameters that are monitored differently according to gender, because the surgery to be performed may contribute to a feminine appearance of the face.

The reason for the lack of a significant relationship between age groups and parameters may be the age range of the sample group used in our study. In order for age-related changes in mandibular morphology to become more evident, individuals with higher bone resorption should be examined, especially in older age groups. Since edentulism and bone resorption status were not taken into consideration in our study, it was not possible to fully evaluate the effect of this variable. In addition, since our study was retrospective, it could not be determined whether the patients had bruxism. In future studies, multicenter studies should be conducted in individuals with different ethnic backgrounds, by increasing the subgroups of age groups, supported by 3D shapes, and examining the effects of diseases affecting the muscles such as bruxism.

## 5. Conclusions

Our findings suggest that the male mandible may have a longer condyle, a narrower mandibular angle, and a deeper sigmoid notch than the female mandible. It has been observed that ramus measurements such as the gonial angle, condyle length, condyle–coronoid distance and sigmoid notch depth may be important in gender discrimination. These findings may provide clinicians with useful information in gender determination, orthognathic planning, and jaw surgery planning. Although differences were observed in the parameters examined in our study, larger, multicenter, and ethnically diverse studies are needed to reliably apply these parameters in forensic settings. When the gonial angle, coronoid height, sigmoid notch depth, condyle height, and condyle–coronoid distance were examined between age groups on both sides, no significant difference was found. However, values may differ in individuals with different ethnic origins in different regions. Therefore, the relationship between age and gender of the parameters should be investigated with more comprehensive studies in different ethnic origins.

## Figures and Tables

**Table 1:** Distribution of measurements by gender.

	Gender	*N*	Mean	Std. Deviation	*p*
Right gonial angle	female	78	128.6615	5.50129	0.001
	male	63	125.6841	5.10352	
Right coronoid height	female	78	11.1654	2.94560	0.852
	male	63	11.2714	3.63120	
Right sigmoid notch depth	female	78	13.1295	1.79943	0.044
	male	63	13.8841	2.46283	
Right condyle height	female	78	16.2769	3.32624	0.006
	male	63	17.8571	3.36527	
Distance between right condyle and coronoid	female	78	33.9244	3.26040	0.007
	male	63	35.5222	3.60392	
Left gonial angle	female	78	128.8364	5.97237	0.000
	male	63	125.2635	4.89326	
Left coronoid height	female	78	10.8923	2.68167	0.220
	male	63	11.6095	3.92879	
Left sigmoid notch depth	female	78	13.1397	1.61054	0.020
	male	63	14.0111	2.55369	
Left condyle height	female	78	16.4885	3.38115	0.025
	male	63	17.7635	3.25697	
Distance between left condyle and coronoid	female	78	33.8885	3.38257	0.101
	male	63	34.8476	3.46921	

**Table 2:** Comparison of age groups.

	*N*	Age Groups					*p*
		20–29	30–39	40–49	50–59	60 and over	
		30	30	24	30	27	
Right gonial angle	(Mean)	126.765	126.450	128.108	127.863	128.300	0.629
	(SD)	5.8384	5.3092	6.5360	5.5068	4.9373	
Right coronoid height	(Mean)	11.0921	11.6735	11.0250	11.1400	10.3000	0.141
	(SD)	3.2874	3.0329	4.4634	2.9019	3.0775	
Right sigmoid notch depth	(Mean)	13.4026	13.7206	13.0500	13.1867	12.6222	0.072
	(SD)	1.8919	2.4210	2.2581	2.1071	1.9146	
Right condyle height	(Mean)	16.9737	16.5000	17.8750	17.5400	16.5889	0.625
	(SD)	3.8518	3.3181	3.4431	3.3255	3.0806	
Right condyle–coronoid distance	(Mean)	33.5263	34.7353	35.7667	35.4700	34.6556	0.145
	(SD)	3.7899	3.5671	1.4022	3.8768	2.9127	
Left gonial angle	(Mean)	126.252	126.717	128.316	127.833	129.276	0.270
	(SD)	5.8411	6.4570	6.0936	5.2597	4.9626	
Left coronoid height	(Mean)	11.1711	11.9294	10.4500	11.7800	10.0778	0.176
	(SD)	3.4463	3.1343	4.9458	3.0573	2.4489	
Left sigmoid notch depth	(Mean)	13.4184	14.0206	13.3333	13.9833	12.6481	0.087
	(SD)	1.9656	2.2569	2.3784	2.2028	1.7566	
Left condyle height	(Mean)	16.9579	16.9706	17.7000	16.8800	16.7778	0.536
	(SD)	3.5679	3.5178	3.1343	2.9202	3.5290	
Left condyle–coronoid distance	(Mean)	33.2526	34.4147	34.7500	34.2100	34.0630	0.093
	(SD)	3.3842	3.5865	2.6729	3.5747	3.2117	

*N*: number; SD: standard deviation.

## Data Availability

The data presented in this study are available on request from the corresponding author due to (the reason for the restriction is because the study contains personal data).
